# Physicians' Ineffective Communication Leading to Cerebral Injuries in Children With Cerebral Palsy

**DOI:** 10.7759/cureus.29510

**Published:** 2022-09-23

**Authors:** Suliman Elwagei Ahmed

**Affiliations:** 1 Pediatrics Department, The National Ribat University, Khartoum, SDN

**Keywords:** communication, intracranial hemorrhage (ich), asphyxia, aspiration pneumonia, cerebral palsy (cp)

## Abstract

Inspirational enlightenment has guided me to write this review article while encountering, during my practice as a pediatrician, referred cases of previously stable, positively progressing cerebral palsy (CP) children who had not yet celebrated their first teen birthday but ended up being in a deep coma and mechanical ventilation-dependent post a cardiac arrest outside the hospital. The dramatic end was believed to be probably caused by food aspiration, which could have been prevented by effective counseling to the in-denial, struggling parents about their children’s condition. This report tries to emphasize the importance of effective communication between physicians and caregivers of children who were diagnosed with CP. The importance is elaborated by linking the level of the provided communication/education to the caregivers with cerebral injuries, such as intracranial hemorrhage (ICH), which potentially could have been prevented among CP children. The sequence of the injury is believed to be initiated by aspiration of food, which resulted in apnea/asphyxia, followed by a cardiopulmonary arrest outside hospital settings. Such a life-threatening event is hypothesized to be the leading cause of non-reversible intracranial injuries to the CP child of misinformed/unaware parents. Data on unsolved parental status, parent-provider miscommunications, and aspiration pneumonia leading to cerebral injuries and their permanent neurological insult in CP children were reviewed.

## Introduction and background

An internationally agreed cerebral palsy (CP) definition states that it is a collection of constant posture and motor disorders, leading to restriction of activity, which is caused by non-progressive disruptions that take place in the growing fetus's brain or in the not fully matured. The movement deficiencies of CP are generally associated with disorders of perception, cognition, sensation, behavior, and communication, as well as seizure disorder and derivative musculoskeletal consequences [1].

Stavsky et al. mentioned that the incidence of CP worldwide is two to three per 1,000 live births, and low birth weight and prematurity are important CP risk factors [2]. Stavsky et al. also reported that various other factors are associated with an increased risk of CP, for instance, multiple gestations and maternal infections.

Similar to the Patel et al. study, Germany et al. acknowledged the fact that the majority of CP cases are a result of damage to the brain of the fetus or the neonate; they found that the onset of CP in the post-neonatal period has been known to occur prior to 24 months but after 28 days of age [1,3]. The most common reasons in this age group are trauma to the brain, meningitis, and vascular event/near-drowning [1,3].

Agreeing with the Patel et al. study, Sankar et al. stated that CP can present clinically in a different spectrum, yet is generally classified as hypotonic, spastic, dyskinetic, or mixed [1,4]. Patel et al. mentioned that during the first two years of a child's life, it is difficult to make, with certainty, a specific diagnosis of CP in most primary care or pediatric practice settings since it, in many cases, has no apparent cause. However, the diagnosis is based on meticulous clinical history, findings on MRI scans, and a standardized neuromotor assessment; precisely, if we know that it is associated with oro-motor problems, that could be life-threatening without early intervention. Patel et al. estimated that 50% of infants who have CP have recognizable high-risk factors, which permits early detection and diagnosis [1]. Those infants, without any recorded risk factor at birth, first come for medical advice when their parents suspect delayed or unusual developmental progression of the neuromotor [4].

It is understood from the scientific context that a physician would be suspicious of CP upon a significant delay in the milestones of a child, motor or cognitive, for example, and in correlation with each individual's prenatal, perinatal, postnatal, and infancy history. While addressing the invariability of CP association with other deficits at first presentation, it should be explained to the parents that CP etiology might not be recognized based on the findings of the two studies of Patel et al. and Sankar et al. Yet, it is crucial to undergo neurodevelopmental screening for a suspected CP child since a prompt intervention program would be of benefit [1,4].

## Review

Aspiration pneumonia

Aspiration pneumonia is fatal, according to a very recently published book, which is conclusive about studies of aspiration pneumonia that were conducted mainly in the US. The researchers found that initially hospitalized patients with community-acquired pneumonia have a risk of developing aspiration pneumonia of around 13.8%, and its rate of mortality is up to 70%, depending mainly on the content and volume of aspirate [5].

Aspiration pneumonia is the main, possibly negative sequel that happens in patients with advanced cerebral palsy and is correlated with their existing prognosis, requiring adequate evaluation and intervention [1,6]. Since the frequency of swallowing can be a reliable indicator for estimating the risk of pneumonia in those severely affected cerebral palsy patients, as shown in a study that included 57 patients with CP. The frequency of swallowing was calculated for every individual three times on different days, inspecting the relationship between the occurrence of pneumonia and the swallowing frequency by applying an analysis of the logistic multivariate regression. It concluded that swallowing frequency was constant within individuals (with a positive correlation coefficient: 0.941). Additionally, the frequency of swallowing was at an hourly rate of 27.0 ± 20.4 and 12.2 ± 12.2 in groups of patients who didn't or did have pneumonia in the past, respectively (P < 0.001) [6].

Such findings raise a concern that a lower capability to swallow is associated with a higher incidence of aspiration pneumonia among CP patients, or even further, the inability to swallow in a CP child is a 100% guarantee to develop aspiration pneumonia in the case of non-medically justified oral feeding.

Çakar and Cinel (2021) conducted a Turkish study of 83 CP patients who were hospitalized 292 times [7]. They questioned the respiratory-related issues in CP patients that permits hospitalization; they found pneumonia coming after chronic aspiration is considered the highest incidence among complications of the pulmonary system in CP patients, and chronic respiratory failure is the anticipated consequence. The study also mentioned that patients who were admitted three or more times during the oral feeding period had higher total hospitalization and ICU stay frequency/duration [7]. The study necessitated assessing such patients by looking for difficulties in feeding at an early stage with suitable diagnostic tools, for example, video-fluoroscopy, and hence, finding solutions before the lung's parenchyma becomes affected. These solutions could include interventions such as artificial feeding (nasogastric tube or gastric tube with or without fundoplication), However, they should prevent any frequent lung infections that could precede respiratory collapse [7].

Brain injury and apnea or cardiorespiratory collapse

Although, very limited choices of studies have been found confirming the possibility of causation between the brains of neonates’ anatomical changes or older children’s brains and asphyxia, apnea, or cardiopulmonary collapse. This deficiency was due to copyright or non-open-access policy.

Apnea plays a crucial role in intracranial injuries; this has been proven in a review study that concluded basic and timely interventions to impede apnea could enhance clinical outcomes and mark it as an avoidable contributor to death, which is usually referred to as a crucial injury of the brain [8].

A German study, not too long ago, has concluded that there is a significant difference between resuscitation after traumatic cardiac arrest versus nontraumatic reasons. It is fundamental to treat changeable factors, including hypoxia. On the grounds of hypoxic-cardiac arrest, it should be sorted out by prompt airway intervention and ventilation [9].

In a severe form, hypoxia will eventually change the cerebral hemodynamic structure, which might lead to a cerebral insult either due to an acute event or a congenital cardiopulmonary etiology; this was reported in a 37-year-old male, who was an unusual case of a pseudo-subarachnoid hemorrhage due to chronic hypoxemia [10].

Long-term outcome post intracranial hemorrhage

In Korea, Shin et al. recently conducted a multicenter retrospective cohort study on intracranial hemorrhage (ICH) incidence as a reason for out-of-hospital cardiac arrest (OHCA). This was conducted among 2716 patients over a period of six years. The addressed OHCA cases were evaluated for a comparison between the ICH and non-ICH groups, and the study noted that ICH was the underlying factor of cardiac arrest in 92 patients (11.4%) after the return of spontaneous circulation. Within this ICH group, 79 (86%) people developed a subarachnoid hemorrhage as well. No one had a good neurological outcome in the ICH group [11].

A correlation between the low Glasgow Coma Scale (GCS) post-ICH and a high mortality rate has been found, as per a Chinese single-center observational study, which was run between 1996 and 2010. It targeted detecting the long-term sequences of cerebellar hemorrhage that occurs spontaneously; from 440 included participants with primary ICH, 16.4% (72 patients) had cerebellar hemorrhage as a primary, aging between 55-80 years old, with a mean age of 67.5 years. The one-month death rate was around 16%, with GCS ≤ 8 as an orphan predictor. After six months, around 56% of patients who lived after the initial 30 days, had a poor functional status that persisted with a modified Rankin scale score >2 [12]. This correlation is consistent with the profound functional disability post-ICH that was mentioned above in the study of Shin et al.

Generally, it is understood that cerebral palsy, not to mention a complicated case with ICH, has no cure, but a multidisciplinary intervention would benefit the participation, functionality, and life quality of the patient. The management involves the child's family and should always be applied in terms of family needs, values, and capacity [13].

Parental reactions to cerebral palsy

A cross-sectional study in the Netherlands was conducted on 51 parents of CP children as part of an already established longitudinal research program. It found in one-third (23%) of the cases, unlike other cited studies with a higher proportion (44-59%), that the “unresolved” status of parents was associated with the severity of CP; the limitations of their child's gross motor skills seemed to symbolize a leading factor demonstrating negative parental reactions such as avoidance, denial, or escape. Despite the acknowledged limitations within this particular study, new perspectives have been served in terms of parents' resolution status, i.e., unresolved feelings and thoughts regarding the caregiving of a CP child could intervene with the child's comfortable interaction by impacting the comprehension of children's tolerance and needs [14]. This overlooked scene has a key role in determining the prognostic outcome of a CP child with an unresolved/denied state of mind of the parents. Without providing appropriate counseling, multidisciplinary support, and regular follow-ups, God forbid, a bite of food could be the child's last.

In 2009, a study based on the Interview of Reaction to Diagnosis, in a clinic setting, evaluated 255 parents of CP children aged between 1.4 and 17.3 years. The predominant responses (81.6%) indicated resolution. The study was significantly clear that unresolved status was found among younger children's parents and those who are parenting children with severe forms of motor disabilities. According to the study, resolution was mainly focused on action to better the child's life among teenagers' parents, in contrast to younger children's parents; unresolved status manifested from parents seeking to focus on more information and constructive ideas [15]. This age-associated status is a clear marker predicting the age groups of CP children who are at risk of misjudgment by unresolved parents, which are CP children before adolescence. This conforms to the scenarios that I encountered with deteriorated pneumonia in CP children due to food aspiration.

Moreover, in Serbia (2015), an observational study of 100 children's mothers who were aged between two and seven years and diagnosed with cerebral palsy, showed that mothers' depression and the functional status of CP children were associated significantly as predictors of the resolution status. The study was based on the Reaction to Diagnosis Interview [16]. This explains, to some extent, the strong relationship between unresolved caregivers' status and the risk of developing an unpleasant event (aspiration pneumonia for example) that might lead to ICH in non-adolescent severely disabled CP children.

Importance of interdisciplinary and support teams

According to Shevell et al. and Jan MM, multidisciplinary integrated care is a need that might predetermine the involvement of neurologists, developmental pediatricians, occupational therapists, physiotherapists, radiologists, orthopedists, ophthalmologists, audiologists, geneticists, and social practitioners; chromosomal and metabolic analyses is not advised as a routine but considered rational if the child is born with dysmorphism, a positive family history of delay, or parents' consanguinity. They further added that brain MRI is more sensitive than CT in highlighting the range of changes in white matter [17,18].

In the early detection of CP, there is no specific protocol to follow, but according to a comprehensive review article by Mohammed MS that is indexed in the NIH, there are red flags for CP, and their presence would warrant an early suspicion by the pediatrician of the diagnosis; additionally, there will be a more beneficial outcome of the early intervention, in terms of improving the quality of the child's and both parents' lives, according to the same revision [18]. Nevertheless, these signs implicate a delay in development, walking on toes, continuously fisting, epilepsy, microcephaly, restlessness, little-to-no suckling, the dominance of one hand before the age of two years (signalizes hemiparesis), and crossing legs. Further, inborn reflex constancy is considered a precocious indicator [18].

An executed qualitative study about "The End-of-Life Care Experiences of Relatives" took place in 20 ICUs among hospitals in the UK by interviewing only 22% (29 of the next of kin of 130 patients who died during the study), who agreed to be enrolled in this interviews-based study. This measuring study concentrated on the noted needs for palliative care of almost 30 families. Assignees cherished the physical care their loved ones had received, but at the same time, they were concerned about breaking bad news and communication as reported. The paper urges training medical staff on how to deliver bad news, as it is evident that the input of a team of palliative care professionals would be of worth and plays a significant role in facilitating the communication of information to families regarding devastating neurological complications, including the death of the brain. Also, it concluded that taking into account adding other palliative care members will support the parents/relatives while passing through this traumatic time as well as the process of bereavement [19].

Miscommunication

According to a Harvard-based pediatrician' study led by Alisa Khan et al., parent-provider miscommunications in hospitalized children are related to a suboptimal hospital experience and parent-reported errors [20]. It could also lead to lengthening the intra-hospital stay in some cases, as seen in this cohort study of more than 470 parents of children, aged from their first day of life up to their seventeenth year.

Preventable faults in communication with the parents of ill children, generally, and of children with CP, especially, in terms of approach, timing, or environment are ultimately the responsibility of the attending physician, who may not be as involved in the day-by-day care of patients as nurses or residents because he or she would not be overseeing all miscommunications that occur with parents. However, the attendant is the main person responsible for patient care, security, or malpractice-related effects that could arise from miscommunication in the patient's care team [20].

A systematic review with a meta-synthesis from the Netherlands was published in 2021. It studied the effects of communication between 693 providers and 6960 parents during the hospitalization of their infants in 300 NICUs. The impactful effect of communication was reported, distinctly in the reduction of the anxiety and stress of the parents. The study concluded that healthcare providers must consider the impact on parents of their daily interactions, as it is a critical determinant for the comfort of the parents and their contentment with the level of the provided care [21]. Likewise, the level of importance in terms of effective communication with the parents of children who are admitted to PICUs, not to mention a life-changing complication of those known to be CP.

Another qualitative study, conducted in 2021, laid stress on a fixed analytical comparison of touching-ground interviews of 64 parents, who were or were not bereaved, of 44 children (aged from one to 12 years, 61% passed away) diagnosed as having a life-threatening disease. The results render practical indices on how bad news delivery should be improved. Based on the experiences of those parents, the following 10 points obstructing the communication of unpleasant news were formulated: (1) an insufficiency of communication (in terms of timing), (2) failure of physicians to request parents' feedback, (3) parents' sense of unpreparedness within and post-conversation, (4) shortage in clarity regarding treatment for the future, (5) failure of physicians in expressing uncertainties, (6) failure in scheduling conversations as follow-up by physicians, (7) redundancy in numbers of unidentified healthcare professionals, (8) concerns by parents of how to break the bad news to their young ones, (9) determining the significance of bad news in non-verbal circumstances, and (10) misapprehension of medical terms by parents [22].

Kim et al.'s study confirms the non-promising long-term neurological outcome for patients who experienced ICH, which is associated with OHCA [11]. Also, it highlights the considerable risk of developing cardiac arrest due to ICH, which probably would be provoked by hypoxia, that is resulting from silent food aspiration, apnea or so in CP patients.

During our management of these specific complicated cases of cerebral palsy children, a suspicion of a new onset seizure disorder was confirmed by an EEG study in one aspiration pneumonia child, who had a sequence of OHCA and then ICH. The test was done after a persistent high reading of blood pressure and tachycardia. This patient was in a deep coma and mechanically ventilated, and her/his condition persisted despite the establishment of restrictive infused fluids measures and furosemide medication. Such findings were reported during the past few years in a leading European country by a highly specialized care center that conducted a study on the long-term complications of atraumatic subarachnoid hemorrhage (SAH) patients. The target sample, mortality rate, percentage of resultant epilepsy, and other complications cannot be relayed in my review due to copyright restrictions. However, the study reported the occurrence of these events as long-term complications in a frequent trend of post-non-traumatic SAH, which were accompanied by impairment of social and functional outcomes. In my perspective, this urges attention to the fact of non-promising long-term neurological outcome that was reported by Kim's study as well.

Appropriate communication

Such aforementioned obligations of the treating consultant, i.e., attendant, in the Khan et al. report, necessitate an extra effort while communicating with the pediatrician about all types of patient families and CP children in specific [16]. Counting on such proactive and protective measures would improve the level of effective communication and decrease the detrimental consequences that might result from the opposite. However, it is recognized that such a corrective process will need continuity and long-term follow-up with the families of CP children, which puts a burden on pediatricians and the resources of facilities.

On most occasions, parents are eager to know the severity of their children's cerebral palsy and if they will be able to regain basic motor activity, e.g., eating or walking. Usually, similar questions are difficult to answer, particularly for a non-familiar physician with such a condition. Revising literature on disclosure of truth and communication calls for honesty, frankness, consulting both parents at a time, and the discretion of each family's sensitivity to individual needs. This depicts the need to share all the circumstances involving the CP child’s condition with the parents. While cautiously and sympathetically delivering important information, it should be compatible with breaking bad news ethics. Little do we know that a concise counseling session with a single phrase such as "no hope", "it is impossible", "it will not”, etc. would add insult to injury. Especially for denial-unresolved parents, their reactions to such a shocking attitude are extremely unpredicted.

## Conclusions

CP is an incurable condition, and if complicated with ICH, its neurological long-term prognosis is unfavorable. The incidence of such complications among CP children will need to be identified by further studies, yet its outcome seems devastating for such a fragile group of patients. Since studies show that a good percentage of CP children’s parents have an unresolved status with the diagnosis of cerebral palsy, it becomes necessary for physicians to communicate effectively in a long-lasting fashion and to put in extra efforts while being enrolled alongside other healthcare providers in effective communication courses. Additionally, following up should remain strictly under a multidisciplinary approach for all CP children.
